# Plasma Exchange in Severe Attacks of Neuromyelitis Optica

**DOI:** 10.1155/2012/787630

**Published:** 2012-02-12

**Authors:** Mickael Bonnan, Philippe Cabre

**Affiliations:** ^1^Service de Neurologie, Hopital F. Mitterand, 64000 Pau, France; ^2^Service de Neurologie, Hôpital Zobda Quitman, 97261 Fort de France, Martinique

## Abstract

*Background*. Neuromyelitis optica (NMO) attacks are poorly controlled by steroids and evolve in stepwise neurological impairments. Assuming the strong humoral response underlying NMO attacks, plasma exchange (PLEX) is an appropriate technique in severe NMO attacks. *Objective*. Presenting an up-to-date review of the literature of PLEX in NMO. *Methods*. We summarize the rationale of PLEX in relation with the physiology of NMO, the main technical aspects, and the available studies. *Results*. PLEX in severe attacks from myelitis or optic neuritis are associated with a better outcome, depending on PLEX delay (“time is cord and eyes”). NMO-IgG status has no influence. Finally, we build up an original concept linking the inner dynamic of the lesion, the timing of PLEX onset and the expected clinical results. *Conclusion*. PLEX is a safe and efficient add-on therapy in NMO, in synergy with steroids. Large therapeutic trials are required to definitely assess the procedure and define the time opportunity window.

## 1. Introduction

Neuromyelitis optica (NMO) is an inflammatory disorder restricted to the spinal cord and optic nerves. Contrary to multiple sclerosis (MS), relapses of NMO are often strikingly severe and most NMO patients present stepwise neurological impairments. NMO treatments are aimed to prevent the relapses with the administration of various promising immunosuppressive drugs. However, relapse treatment is still a tricky problem. Since the largely used steroid treatment usually fails to control severe attacks, specific add-on treatments have to be considered in order to limit the stepwise increase of residual impairment. Given that a strong humoral response characterizes NMO physiology, one might assume plasma exchange (PLEX) to be particularly well adapted in severe NMO relapses. 

We here propose to outline the rationale of the PLEX treatment based on physiological grounds and summarize the relevant data of PLEX studies in the setting of NMO spectrum disorder, assessing the results obtained in each type of attacks. Finally we will try to build up an original concept linking the inner dynamic of the lesion, the timing of PLEX, onset, and the expected clinical results.

## 2. Physiopathology of NMO

### 2.1. Pathology of NMO Lesions

A characteristic pathological pattern has been described in NMO [1]. Lesions are infiltrated by neutrophils and eosinophils and wall capillaries are hyalinized. A vasculocentric pattern of activated complement and immunoglobulin of IgG and IgM types is observed that mirrors the normal expression of AQP4. AQP4 expression is definitely reduced in normal appearing white matter and lost throughout the lesions. These modifications are the hallmark of NMO and could occur alone or associated with a wide range of lesions from mild demyelination to large necrosis. This pattern of lesion was classified in the pattern II of the Lassmann classification of the inflammatory lesions [1, 2]. Contrary to MS, T cells are rare in NMO lesions and probably have no major effect on the formation of the lesions [3]. However, T cells are probably involved upstream in physiopathological cascade in the earlier phases of the disease where a complex interplay leads to antigen sensitization and possibly in the initial opening of the blood-brain barrier (BBB) [4]. Moreover, the pattern associating vasculocentric deposition of Ig and complement, cells (eosinophils/lymphocytes) infiltration and AQP4 loss is sometimes fully dissociated from demyelination [5]. 

#### 2.1.1. Specific Antibodies and Their Epitopes

The NMO-IgG antibody is IgG1 directed against protein aquaporin-4 (AQP4) [6]. This antibody is detected with tissue-based immunofluorescence assays with a sensitivity and specificity for clinically defined NMO of more than, respectively, 60% and 90%. Clinically diagnosed NMO patients share clinical common and evolutional characteristics regardless of their NMO-IgG status. Beyond the surrogate marker value of NMO-IgG, this marker is now used as a major diagnostic criterion [7] and delineates the *NMO spectrum disorders* that gather in a same entity both typical NMO and unusual or truncated clinical forms [8].

AQP4 is a transmembrane protein expressed in the apical domain of the membrane feet expansions of the astrocytes close to the surrounded blood vessels. They are generally found as single tetramers, closely arranged in orthogonal arrays. This protein is critically involved in the homeostasis of the water in the brain and interfaces with blood vessels, especially in the clearance of free water. Loss of perivascular AQP4 in the basal state results in cellular swelling, ostensibly due to a failure to eliminate water generated from cellular metabolism [9]. Thus in NMO, since the interaction of NMO-IgG and AQP4 leads to a functional knockout phenotype of AQP4, edema develops as a result of functional impairment of AQP4 although BBB is expected to be still intact, which may explain the paradoxical lack of gadolinium enhancement in most NMO lesions. Apart from water homeostasis, the removal of AQP4 from astrocytes membrane is associated with an impaired homeostasis of glutamate via the loss of function of EAAT2, a major glutamate transporter associated with AQP4 in a macromolecular complex [10]. The disruption of glutamate homeostasis initiates an excitotoxic mechanism damaging oligodendrocytes and ultimately leading to demyelination [11].

Virtually all the CNS astrocytes express AQP4, however, some regions are enriched in AQP4. Those regions are the spinal cord gray matter, the posterior optic nerve, the floor of the fourth ventricle and the circumventricular organs especially the area postrema, explaining the restriction of the sites of lesion characterizing NMO [12]. Interestingly circumventricular organs are also the only sites of the CNS expressing fenestrated capillaries favoring local passive diffusion of circulating antibodies. 

#### 2.1.2. NMO-IgG and Complement as Key Factors

Clinical activity may correlate with the underlying NMO-IgG titres. NMO-IgG detection is a strong predictor of recurrence after an initial spinal or optic attack [13–15]. In few patients, NMO-IgG was high during flares and became negative during the stabilized disease following treatment, and, in contrary, an initially seronegative patient became positive during a further attack [16, 17]. That is to say that NMO-IgG negative sera are not always NMO-IgG negative patients on long term. In the seminal work of Takahashi et al. [18], NMO-IgG levels were positively correlated with both clinical severity (i.e., blindness) and radiological severity. Moreover, a strong positive correlation was obtained between the NMO-IgG titres at the nadir of exacerbations and the spinal cord lesion length on MRI [18]. In contrast, low NMO-IgG titres were observed during remission induced by immunosuppressive maintenance therapy [14]. 

In vitro, the binding of NMO-IgG to the extracellular domain of AQP4 reversibly downregulates its plasma expression. In the presence of active complement, this binding leads to strong complement activation and rapid cell destruction. NMO serum IgM is not AQP4-specific and abundant IgM deposits in the NMO lesions may have passively diffused after the BBB disruption by the seminal focal complement activation initiated by NMO-IgG [19]. 

In an animal model of EAE with passive transfer of NMO-IgG, the transfer exacerbated EAE signs and the typical pathological characteristics were reproduced in treated rats [20, 21]. Direct injection of NMO-IgG in mice brains could reproduce the pathology, but only when complement is coinjected [22].

The NMO-IgG ability to lesion AQP4-transfected cells in the presence of complement was assessed with serum drawn from patients with mild and severe attacks. The percentage of cells lesioned by complement was strongly higher in presence of sera from patients with severe attacks, although lesion induced by sera from patients with mild attacks did not differ from negative controls or MS patients [23]. Thus, the severity of the disease may be partly determined by intrinsic NMO-IgG characteristics to activate the complement.

### 2.2. Proof of Concept of PLEX in NMO

As we already described, NMO lesions are associated with a strong IgG, IgM and complement deposition, typical of the pattern II in the Lassmann classification. The NMO-IgG is involved in a complement-dependant toxicity against the astrocytes. All of these components—IgG, IgM, and complement—are targeted by plasma exchanges. By means of 5 exchanges, all the exchanged molecules will drop to less than 20% of their initial level [24, 25]. By this way, antibodies and complement, which are the core of the pattern II lesions, are excluded from the circulating pool and cannot migrate anymore to the lesions. 

Although PLEX has long been used in various demyelinating disorders [26], there is some clue that the pattern is a key determinant of PLEX efficiency. In a retrospective study, Keegan et al. [27] reported that all the patients suffering from demyelinating disorders and improved by PLEX had a biopsy proven pattern II lesion. None of the patients with any other kind of lesion improved. However all these patients were MS without NMO-IgG and none were NMO [28]. 

All the aforementioned findings stress that circulating NMO-IgG and complements are the two main actors of the NMO pathogeny and why clearing them from blood with PLEX should be appropriate for special benefits.

## 3. Plasma Exchange Procedure

### 3.1. Principles and Goals

PLEX or plasmapheresis is the filtration of the plasma, which is removed, replaced by artificial plasma and reinfused to the patient—*the plasma exchange*. Basically, the goal of the filtration step is to remove a given volume of patients plasma and to return an artificial plasma substitute in its place [24]. 

### 3.2. Technique

PLEX is carried on in a nephrology or a resuscitation ward. Two high flow rate accesses are mandatory: an input line from patient to device (“artery”) and a return line from device to patient (“vein”). In continuous filtration, two needles are placed in both arms or groins in order to drawn out the blood of the body through an extracorporeal line connected to one needle, then blood is processed and reinfused continuously through the other needle. In case of discontinuous filtration, the separation and remixing are done in small batches through a single venous access in the groin where in and out cycles may alternate. Anticoagulation (citrate or heparin) is added to the blood preplasma filter to prevent from clotting. The removed blood is processed (*apheresis procedure*) in a cell separator that continuously separates plasma from cellular components (consisting of red and white blood cells and platelets) either by a centrifugation ring with permanent in and out flow, or by filtration through a porous membrane. Small molecules like cytokines as well as large molecules, such as albumin and immunoglobulin, are easily extruded from the blood compartment with a reported sieving coefficient >0.95 at a blood flow rate of 100 mL/min. The cleared cellular components are then combined with the replacement fluid (donor plasma or artificial albumin mixed with a saline solution) and returned to the patient through the needle in the other arm. A PLEX session is usually performed in 2 to 6 hours, depending on patient's height, weight, viscosity of the blood, and technical parameters.

### 3.3. Kinetics of the Target Exchanged Components

All the targeted components are distributed in the interstitium (extra-vascular compartment) by variable part. Large molecular weight compounds equilibrate slowly between the vascular space and the interstitium. The relation curve of the achieved concentration of a plasma component (C) after a unique exchange of a given plasma volume is exponential inverse, following a single kinetics. The larger volume of plasma exchanged during each session clears a larger amount targeted circulating component. An exchange of one body plasma volume leads to the immediate clearance of 50% of the circulating component. A 1.3 body mass volume exchange that removes about 72% of the concentration is generally agreed. Beyond, the volume to process increases massively for too little gain. 

However, according to the distribution of C in the interstitium, the achievement of the clearance of C will necessitate the use of multiple PLEX sessions separated by the time necessary for the equilibration of C concentration between interstitium and vascular spaces. The number and frequency of sessions should be evaluated according to the biological characteristics of the components to remove (synthesis level, vascular distribution, diffusion ability). An empirically driven number of 4 to 6 sessions is usually scheduled. The durability of the immunomodulatory effect after PLEX is difficult to assess and will depend on the turnover rate of the targeted humoral components. Concomitant intensive immunosuppressive therapy (i.e., steroids, mitoxantrone, mycophenolate mofetil, and rituximab) will be required to sustain the obtained depletive effect.

### 3.4. Risks and Side Effects

PLEX are contraindicated in case of ongoing infectious disease, precarious hemodynamics, and active hemorrhage (heparin). Immediate side effects are related to the extracorporeal line: hemodynamic instability, vasovagal syndrome, numbness or tingling, venous puncture hazards with excessive local bleeding, septicaemia, or allergy. Since blood coagulation factors are all depleted by PLEX, hemostasis is affected in variable ways: first, a hypocoagulation state is immediately achieved by the global depletion of all the coagulation factors for half a day; at day 2, short-life procoagulant factors are regained but antithrombin-III synthesis is delayed leading to a hypercoagulable state until day 3. Preventive, anticoagulation with heparin is always required since the high risk of thrombosis. Persistently low fibrinogen levels have been described with the concomitant use of high dosage steroid infusion. In summary, PLEX is generally well tolerated and now commonly and safely used.

### 3.5. PLEX and Steroids

Methylprednisolone pharmacokinetics is characterized by a very short half-life (about 2 hours) and although steroid binding to plasma protein is about 75%, the volume of distribution is very large (about 1.5 L/kg) [29]. These factors are the key of the negligible PLEX effect upon steroids biodisponibility [30, 31]. Of note, all the relapses received steroids. When used the same day as PLEX procedure, steroids were infused at the end of each PLEX session.

## 4. PLEX in Severe Attacks

Various regimens of high doses of intravenous methylprednisolone are used in first line of treatment ranging from 3 g infused in 3 days, to 10 g in 5 to 10 days, depending on authors. There is no evidence in favor of one regimen or another and efficacy assessment has never been addressed. Moreover, even if steroids reduce the inflammatory cellular response by triggering apoptosis of lymphocytes, they are clearly not sufficient because poor outcomes are still a common issue even when steroid treatment is given immediately after onset. We wish to develop here the evidence for the effectiveness of PLEX that we have been largely using as an add-on therapy for more than 10 years. 

### 4.1. Spinal Attacks

PLEX proved to be efficient in central demyelinating diseases in a randomized sham-controlled study [32, 33]. Keegan et al. [26] reviewed the clinical data from 59 patients who received PLEX for inflammatory demyelinating diseases, including 10 NMO and 6 acute transverse myelitis (ATM) cases. A moderate or marked improvement was obtained in half of NMO and ATM patient groups. The late final outcome at one year was more or less obtained during the first month after treatment in both groups, without regard to success or failure of treatment [26, 34]. A small number of case reports and few small studies were reported with variable issues. Judging improvement is even more complex due to the subjective classification of improvement in mild/moderate/marked instead of a quantified clinical exam [26, 34–36]. Moreover the natural history of single spinal relapse in NMO has never been addressed, so any improvement bias after PLEX is inappreciable in the absence of a control group. Finally, most authors used PLEX as a rescue treatment given late after the onset. For example PLEX was delayed from onset by a mean of 33 ± 30 days in Brunot et al. [34] and a median of 30 days (6 to 90 days) in Llufriu et al. [35].

Although a synergistic effect of steroids and PLEX was long expected due to their complementary action, none of these studies compared conventional steroid treatment alone with add-on PLEX-treated attacks. 

We previously refined these results in a study of outcome after severe spinal attacks associated with NMO spectrum disorders [37]. We included 96 spinal attacks from 43 patients, divided in two groups: (1) a steroid-only group designed from historical patients treated with steroids alone; (2) an active group treated with both PLEX and steroids. Steroid infusion was started immediately after patient admission. PLEX decision was raised at the same time and started as soon as possible during the two days later. As a major difference with other groups, PLEX was never initiated as a delayed rescue treatment after a standard steroid treatment failure. Since PLEX therapy is mainly expected to minimize residual impairment, we used the ΔEDSS (calculated as the difference between residual and basal EDSS) as the main outcome.

If we except 5 PLEX delayed due to difficult medevac reasons, PLEX was initiated by a mean of 5.4 ± 3.1 days after attack onset with a median of 4 sessions. There was no significant difference between the PLEX-treated and steroid-only groups for basal and acute EDSS (3.9 ± 2.9 versus 4.2 ± 2.9, and 7.9 ± 1.0 versus 8.0 ± 1.4; *P* = NS), however, residual EDSS (5.1 ± 2.4 versus 6.8 ± 1.9, *P* < 0.01) and mean ΔEDSS (1.2 ± 1.6 versus 2.6 ± 2.4, *P* < 0.01) were significantly lower in the PLEX-treated group than in the steroid-only group. 

Basal EDSS dramatically influenced therapy outcome (Table 1). During the first attack, although acute EDSS were similar in both groups (7.6 ± 1.2 versus 7.1 ± 1.5, *P* = NS), ΔEDSS and residual EDSS were dramatically reduced in the PLEX-treated group (2.1 ± 1.9 versus 5.9 ± 2.0, *P* < 0.01) given that acute EDSS was similar in this sub-group. In the two other sub-groups of basal impairment (EDSS 1.0 to 5.5 and EDSS ≥ 6.0), residual EDSS and ΔEDSS tended to be lower in PLEX-treated attacks but no statistical signification could be obtained due to the small size of these groups. 

The classical Lazarus effect, defined as a very short-term dramatic improvement [38], was rather unusual in our group but our study was not designed to analyse short-term improvement. The patients who experienced this effect have all received a very early treatment (less than 2 days). However, in Magaña et al.'s paper [39], patients who exhibited functional improvement did so within a median of 4 days (third PLEX), although a minority (6%) exhibited a delayed response (more than 2 months).

Minor side effects occurred in 24% of PLEX-treated attacks and resulted in PLEX interruption once (84-year-old patient with pulmonary embolism). 

In summary, PLEX-treated patients achieved a significantly better outcome after a spinal attack, especially if PLEX was given during the first attack. The exact effect of PLEX in previously impaired patients should be validated in a larger multicentric cohort. As PLEX proved to be a promising treatment in spinal attacks, it would now be unethical to design a study with a sham-treated control group. 

Predictors of good outcome were studied in a large group of PLEX including 26 NMO patients [37]. The only good outcome predictor was normal or brisk reflexes in acute phase [39]. Surprisingly a short PLEX delay was associated with a good outcome in a first study [26] but had no effect in a second study, although one should remind that median PLEX delay was long (23 days) in this later compared to our group. The same PLEX response rate was obtained irrespective of NMO-IgG serostatus in our cohort and in the Mayo Clinic cohort [39].

As a practical consequence, faced with a patient suffering from a severe relapse, the knowledge of NMO-IgG status should not be required to start PLEX as soon as possible, since PLEX was found efficient in NMO-IgG negative patients.

### 4.2. Optic Attacks

Visual impairment in NMO is very severe. We previously showed that an immediate unilateral blindness occurred in a third of patients after the first optic neuritis (ON), and generally two attacks are sufficient to cause a definitive loss of vision [40]. Few PLEX were undertaken after ON and a quick dramatic recovery is usual as we also observed [41, and unpublished results]. Depending authors, PLEX were used immediately [41] or as a delayed add-on therapy [42–45]. After pooling severe (acute visual acuity < 1/10°) ON patients (either NMO or in the NMO spectrum) from available studies [41, 42, 45, 46] with ours (unpublished results), data were gathered for 39 eyes. PLEX were given in a median of 19 days in patients who recovered a visual acuity more than 1/10° (considered here as a treatment success) but 41 days in treatment failure. A clear effect of PLEX delay was observed since success rate was 8/8 (100%) during the first 11 days, then 4/7 (57%) from days 12 to 22, and 7/13 (53%) from days 23 to 73. Moreover, even when patients recovered, averaged residual VA tended to be lower in delayed PLEX patients. Interestingly, the spontaneous recovery (>1/10°) after severe ON treated by steroids alone was about 40% in our cohort (from [40]), which is very close to the recovery obtained in the two last groups of late PLEX. In conclusion, strong clues support that PLEX change the outcome of severe ON only when they are given early, however broader studies using carefully chosen patients are still lacking to confirm this hypothesis. 

### 4.3. Brain Attacks

Apart from opticospinal attacks, severe brain attacks are described in NMO, especially involving hypothalamus and medulla oblongata. Those lesions are usually severe and associated with blindness, central endocrine disorders, or quadriplegia with respiratory failure. Nonspecific telencephalic lesions are common but are mostly asymptomatic [47]. However, symptomatic lesions involving supratentorial white matter are exceptional and extensive [47]. Even if a favourable outcome after PLEX has been reported in a few severe cases [48, 49], comparative data are still lacking. 

Posterior reversible encephalopathy syndrome (PRES) is an encephalopathy with consciousness and visual disturbances with rapidly reversible changes on MRI consistent with vasogenic edema. PRES are triggered by blood pressure instability or fluid stresses due to various causes. It seems to occur more often than coincidental in NMO patients: 5 out of 70 consecutive NMO-IgG patients evaluated at Mayo Clinic [50]; 2 out of 5 in Hadassah Medical School, Israel [51]. Authors proposed that the autoimmune-mediated disruption of the AQP4 water channel function may predisposes to PRES at comparable levels of acute illness [50]. PLEX was involved as a trigger in one case with a good final outcome [50]. In few cases, PLEX were implemented as curative treatment with an overall good outcome [50, 51].

## 5. Timing of PLEX: Evolving to a Key Concept

Besides knowing PLEX are effective and safe, the central question remains: is PLEX necessary as soon as and as often as possible? Prospective, randomised, multicentre clinical trials would be required to definitively answer the question. For most authors, to date PLEX are considered as an add-on rescue treatment after steroid failure. The European recommendation from EFNS is to start with an early steroid course no matter the severity [52]. Early escalation with PLEX is only recommended after a failure of a second course of steroids, that is to say that PLEX initiation may be postponed for more than a week. 

As we demonstrated before, PLEX efficiency depends on the timing of initiation, ranging from immediate dramatic improvement (the *Lazarus effect*) to no effect according to whether they are given early or very late. We propose to regress to the dynamic of the inflammatory NMO lesions to explain why PLEX efficiency is strongly dependant of the timing of their onset. 

### 5.1. Evidence for Reversible Dysfunction Preceding Irreversible Tissue Loss

As we described above, lesion is the consequence of a cascade of reversible events, susceptible to an external action. One could abruptly divide this cascade into two main points: (1) firstly, the binding of NMO-IgG to AQP4 triggers the functional impairment of astrocytes, quickly completed by a complement-mediated cell destruction; (2) the dysfunction of EAAT2 transporters, as a bystander effect, impairs the clearance of free glutamate which progressively accumulates and initiates an excito-toxic mechanism upon oligodendrocytes, ultimately leading to oligodendrocytes apoptosis and demyelination [11, 53]. This excito-toxicity is reversed in vitro by adding a competitive antagonist of the NMDA receptor [11].

The time sequence of these events was studied in lesions induced by direct mouse brain injection with NMO-IgG and complement [22]. Loss of AQP4 and GFAP, and myelin breakdown were evident 7 h following the injection. The inflammatory cells infiltration became evident later. Within 12 h, axonal injury became prominent. By day 7, axonal loss and dying neurons were evident. Finally, one could suppose that a very early intervention targeting astrocytes dysfunction may prevent the progression to the bystander effect. 

### 5.2. Evidence Supporting an Early Treatment

The influence of treatment delay upon outcome has been addressed in a single study of first ON receiving steroid treatments [54]. The outcomes were both visual acuity and the width of the retinal fibers layer evaluated with optic tomography (OCT). Patients were divided into two groups: one group with a good visual outcome, including a high residual visual acuity and high RFL; a second group with a poor visual outcome in terms of low acuity and low RFL. Very interestingly, the two groups were similar in all the parameters except one: patients with a good outcome received steroids with a lower mean delay after ON onset, by a mean of 1.8 ± 1.1 days compared to  7.8 ± 3.8 days in patients with a poor outcome. This study is the first proof that a delayed infusion of steroids is associated with a poorer outcome. A similar effect of treatment delay, although unknown, should be expected in spinal attacks. 

In spinal attacks treated with PLEX, early initiation of treatment was one out of the predictors of good outcome [26]. In a larger study encompassing attacks from various demyelinating disorders, success rates were stratified by delay: improvement occurred in 83% when given before day 15, but fell to 43 after 2 months [35]. Moreover the dramatically very short-term improvement, called the Lazarus effect [38], is sometimes observed after severe attacks receiving a very early treatment with PLEX and steroids. However, this earliness responsibility on the Lazarus effect remains elusive since no study is available on this rather unusual effect.

### 5.3. Lesion Stages and PLEX Action: “Time Is Cord and Eyes”

**Figure 1:**
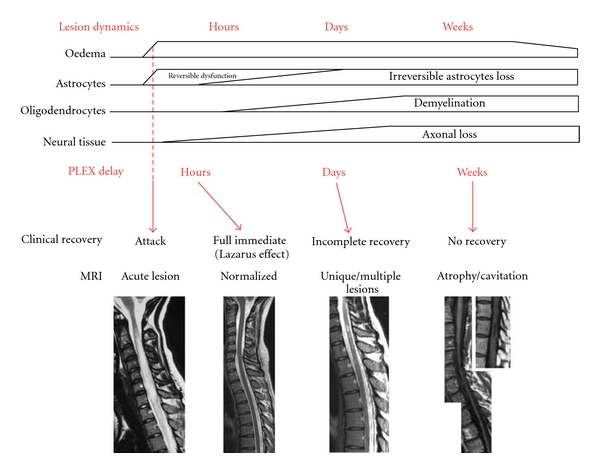
Hypothetical correlation of lesion stages and PLEX effect. The dynamic of the lesion is staged according to pathological data. Clinical and radiological outcome is linked to the delay of PLEX onset.

In the light of the available data, we postulate a link between the staging of NMO lesion and the PLEX effect upon clinical and radiological outcome (Figure 1). *Stage 1 (first hours):* acute attack provokes for hours an astrocyte dysfunction (by NMO-IgG binding on AQP4 leading to internalization) mainly expressed by an edema. This purely edematous lesion could be immediately reversible by the clearance of NMO-IgG preventing the loss of astrocytes and the excitotoxic cascade. Clinical and radiological recovery after PLEX is dramatic and explains the Lazarus effect. *Stage 2 (days):* the underexpression of EAAT2 induces an excitotoxic effect of glutamate on oligodendrocytes leading progressively to demyelination and axonal loss. Astrocytes loss initiates a self-sustained excitotoxic process henceforth independent from NMO-IgG persistence. Even if the extraction of NMO-IgG and complement by PLEX ends the astrocytes aggression. A variable amount of them has been already lost and excitotoxic effects upon oligodendrocytes are evident. Variable amount of tissue is lost as visible on MRI and recovery is incomplete. *Stage 3 (weeks):* atrocytes, oligodendrocytes, and axonal loss are prominent, engulfed in large areas of necrosis. PLEX is almost useless. Neural tissue remains cavitated or atrophic on MRI and no recovery will be expected.

We propose to reconsider PLEX as a major part of the treatment of severe NMO attacks and suggest that PLEX could be given systematically in severe relapses of NMO, extended transverse myelitis or bilateral severe ON resistant to steroids. Moreover, when given they should be started as soon as possible with steroids.

## 6. PLEX as Preventive Treatment

Since NMO-IgG positivity is both predictive of attacks and severity, achieving a low concentration of plasmatic antibodies remains a goal to achieve. Besides immunosuppressive drugs, weekly PLEX have been used to achieve a sustained depletion of NMO-IgG and complement. Favourable cases have been reported but large studies are lacking [55]. Miyamoto and Kusunoki [55] proposed to use PLEX as preventive treatment as an add-on therapy after immunosuppressive drugs failure.

## 7. Preventive Treatments and Future Avenues

The natural history of NMO leads all the patients to a deep impairment in a stepwise fashion without progressive phase. In our study, 5 years after the onset, 70% of patients suffered from a unilateral loss of vision and almost half of them from a bilateral loss of vision [40]. After 8 years, half of the patients had suffered from a severe myelitis and had become chairbound [13]. The mortality rate was very high before immunosuppressive drugs but dramatically dropped since they are largely used [56]. The exact role of recurring PLEX along the remaining attacks to tune the outcome to a low impairment has not yet been addressed but remains most probable considering this striking epidemiological change of mortality in French West Indies. Immunosuppressive drugs are necessary to prevent further relapses but no recommendation is yet available concerning therapeutic escalation.

Since the lesion severity mostly depends on the initial and definitive depth of the loss of AQP4 and astrocytes, future treatments strategies may be directed upon AQP4 preservation. Small molecules or monoclonal antibodies could be used to prevent NMO-IgG binding to AQP4 and to block the physiopathological cascade upstream [57]. Another strategy may deplete pathogenic antibodies by apheresis using dedicated immunoadsorption systems as previously described in myasthenia gravis [58] and in various extra neurological disorders [59]. However, if the value of this technique is less clear in disorders like MS [60, 61], where pathology is broader than a specific antibody, it could be especially suitable to NMO since anti-AQP4 seems to be the pathological core. The strength of these techniques is the specificity of the antigenic absorption with no regard to the class or subclass of antibodies and no fluid replacement [59]. No experience is yet available in the NMO setting. Lymphocytapheresis was successfully described in isolated cases of resistant attacks [62–64]. This alternative therapy may be considered in PLEX-resistant relapses and should be better studied in severe relapses as an alternative of steroids and PLEX. A complementary approach may target the complement system with newly developed anticomplement recombinant antibodies at various levels, with preliminary promising results [22]. Such future treatments may be aimed at preventing or curing the attacks.

## 8. Conclusion

PLEX, in synergy with steroids, could be a major treatment of relapses, aimed at preventing cumulative disability. PLEX is a safe and efficient add-on therapy in NMO. These preliminary results suggest that PLEX may modify the short prognostic of NMO relapses, as long as given early. PLEX proved to be effective regardless of NMO-IgG status. 

Animal models have confirmed that mechanisms leading to lesion evolve over hours and days. Those models should be able to confirm that early therapeutic intervention directed to halt the ongoing lesions should be even more dramatic in an early narrow therapeutic window.

The next steps should be to concentrate upon large multicentric therapeutic trials in order to validate the therapeutic procedure and to determine the time opportunity window. However, we are aware that good trials against placebo could now be difficult to accept since this is an extremely devastating disease.

## Figures and Tables

**Table 1:** Disability measured as EDSS during spinal attacks stratified with basal impairment. St: steroid-only-treated group; *St* + *PE*: steroid- and PE-treated group. Values are given as mean (SD) (from [37]).

	Basal EDSS (null)			Basal EDSS (1.0 to 5.5)			Basal EDSS (≥6.0)		
	St	St + PE	*P*	St	St + PE	*P*	St	St + PE	*P*
	(*n* = 17)	(*n* = 7)		(*n* = 26)	(*n* = 13)		(*n* = 24)	(*n* = 9)	
Basal EDSS	0 (0)	0 (0)	0.99	3.9 (0.8)	3.9 (1.6)	0.59	7.4 (1.0)	7.1 (0.8)	0.52
Acute EDSS	7.1 (1.5)	7.6 (1.2)	0.52	7.6 (1.3)	7.6 (1.1)	0.67	8.9 (0.9)	8.6 (0.6)	0.24
Residual EDSS	5.9 (1.9)	2.1 (1.9)	<0.01	5.8 (1.6)	5.1 (1.1)	0.21	8.5 (1.1)	7.6 (1.0)	0.05
ΔEDSS	5.9 (1.9)	2.1 (1.9)	<0.01	2.0 (1.5)	1.2 (1.6)	0.10	1.1 (0.8)	0.5 (0.8)	0.11
